# Fact vs. fiction: naloxone in the treatment of opioid-induced respiratory depression in the current era of synthetic opioids

**DOI:** 10.3389/fpubh.2024.1346109

**Published:** 2024-02-28

**Authors:** Albert Dahan, Thomas S. Franko, James W. Carroll, David S. Craig, Callie Crow, Jeffrey L. Galinkin, Justin C. Garrity, Joanne Peterson, David B. Rausch

**Affiliations:** ^1^Department of Anesthesiology, Anesthesia and Pain Research Unit, Leiden University Medical Center, Leiden, Netherlands; ^2^Department of Pharmacy Practice, Wilkes University, Wilkes-Barre, PA, United States; ^3^White House Office of National Drug Policy, Washington, DC, United States; ^4^Department of Pharmacy, Moffitt Cancer Center and Research Institute, Tampa, FL, United States; ^5^Drews 27 Chains, Nocona, TX, United States; ^6^Rocky Vista University, Greenwood Village, CO, United States; ^7^Healing Transitions, Raleigh, NC, United States; ^8^Learn to Cope Inc., Taunton, MA, United States; ^9^Tennessee Bureau of Investigation, Nashville, TN, United States

**Keywords:** opioid, opioid-induced respiratory depression, naloxone, fentanyl, xylazine, nitazene, drug abuse

## Abstract

Opioid-induced respiratory depression (OIRD) deaths are ~80,000 a year in the US and are a major public health issue. Approximately 90% of fatal opioid-related deaths are due to synthetic opioids such as fentanyl, most of which is illicitly manufactured and distributed either on its own or as an adulterant to other drugs of abuse such as cocaine or methamphetamine. Other potent opioids such as nitazenes are also increasingly present in the illicit drug supply, and xylazine, a veterinary tranquilizer, is a prevalent additive to opioids and other drugs of abuse. Naloxone is the main treatment used to reverse OIRD and is available as nasal sprays, prefilled naloxone injection devices, and generic naloxone for injection. An overdose needs to be treated as soon as possible to avoid death, and synthetic opioids such as fentanyl are up to 50 times more potent than heroin, so the availability of new, higher-dose, 5-mg prefilled injection or 8-mg intranasal spray naloxone preparations are important additions for emergency treatment of OIRDs, especially by lay people in the community. Higher naloxone doses are expected to reverse a synthetic overdose more rapidly and the current formulations are ideal for use by untrained lay people in the community. There are potential concerns about severe withdrawal symptoms, or pulmonary edema from treatment with high-dose naloxone. However, from the perspective of first responders, the balance of risks would point to administration of naloxone at the dose required to combat the overdose where the risk of death is very high. The presence of xylazines as an adulterant complicates the treatment of OIRDs, as naloxone is probably ineffective, although it will reverse the respiratory depression due to the opioid. For these patients, hospitalization is particularly vital. Education about the benefits of naloxone remains important not only in informing people about how to treat emergency OIRDs but also how to obtain naloxone. A call to emergency services is also essential after administering naloxone because, although the patient may revive, they may overdose again later because of the short half-life of naloxone and the long-lasting potency of fentanyl and its analogs.

## Introduction: the current face of the opioid epidemic

**Figure 1:**
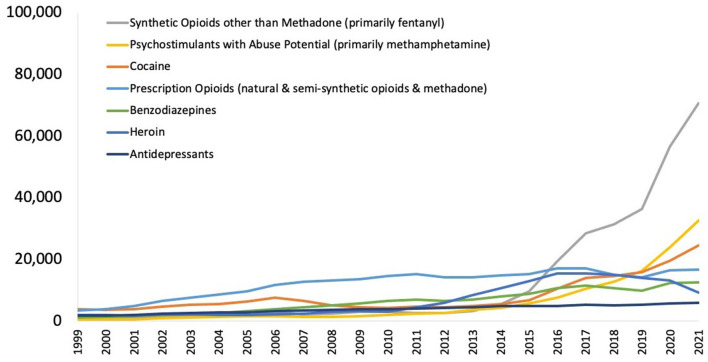
National drug-involved overdose deaths^*^ 1999–2021. From National Institute on Drug Abuse (13). ^*^Includes deaths with underlying causes of unintentional drug poisoning (X40–X44), suicide drug poisoning (X60–X64), homicide drug poisoning (X85), or drug poisoning of undetermined intent (Y10–Y14), as coded in the International Classification of Diseases, 10th Revision.

**Figure 2:**
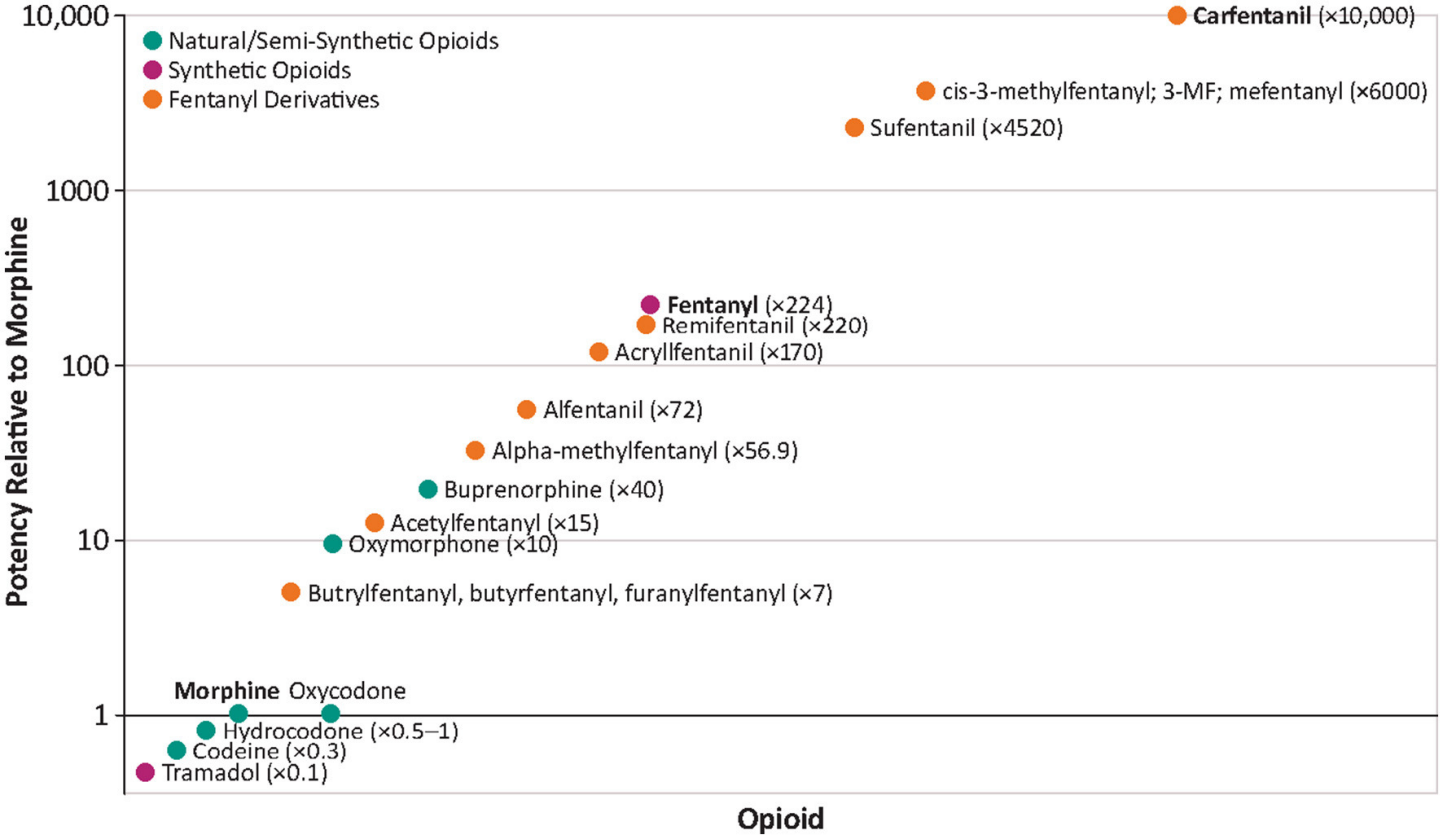
Potency of selected opioids relative to morphine. Data derived from Concheiro et al. (3). ^©^2018 Concheiro, Chesser, Pardi and Cooper, an open-access article distributed under the Creative Commons Attribution License (CC BY).

The total deaths from opioid-induced respiratory depression (OIRD) in the US in 2022 was ~80,000 (1), comprising about three-quarters of all drug overdose deaths. This represents an ~400% increase over the past decade. Most of this increase can be attributed to the exponential growth in the prevalence of synthetic opioids, primarily fentanyl and its analogs such as carfentanil (Figure 1) (2). These compounds have much greater potency than traditional opioids such as heroin and morphine. Pure fentanyl, for example, has 224 times greater potency than morphine, increasing the likelihood that users will experience respiratory depression and fatal hypoxia (3) (Figure 2). In 2021, the nationally age-adjusted drug overdose death rate was 32.4 per 100,000, and the states with the highest rates were West Virginia (90.0) and the District of Columbia (63.6). The states with the lowest death rates were Nebraska (11.4), South Dakota (12.6), and Iowa (15.3) (4).

Opioid-related deaths occur in people with an opioid use disorder (OUD), but may also occur in patients without an OUD using prescribed opioids, particularly if using high doses of daily opioid medications (>50 morphine mg equivalents per day) or using opioids with interacting drugs such as benzodiazepines, gabapentinoids, or alcohol. Patients using prescription opioids in high doses to control pain are at risk of an accidental overdose owing to misreading of instructions, self-medication for additional symptoms, confusion due to age-related cognitive changes, or other factors not directly related to a substance use disorder (5, 6). In 2021, 21% of all OIRD deaths involved prescription opioids (1). A 2017 study of individuals in a Medicaid program who had died from OIRD found that 61.5% of individuals had received chronic non-cancer pain diagnoses in the past year (7, 8). There are also a growing number of accidental overdoses in opioid naïve individuals that occur when the person believe they are taking an otherwise harmless drug but receive one that has been adulterated with an opioid (9). Opioid-related deaths vary across demographic groups and subsets; for example, men have higher overdose rates than women among all age groups and racial and ethnic groups (10, 11) and people who are male, younger, white, and born in the US (compared to those born in another country) are more likely to have an OIRD (12).

## Emergence of synthetic opioid analogs

Synthetic opioids now account for the majority of opioid-involved deaths (2). In 2021, ~90% of all fatal drug OIRDs were due to synthetic opioids (13). The overdose death rates for fentanyl increased by 279% from 2016 to 2021 (from 5.7 per 100,000 to 21.6 per 100,000) (17).

Opioids bind to μ-, κ-, and δ-opioid receptors in the central nervous system (CNS) and peripheral nervous system (18, 19), with μ-opioid receptors being the primary mediators of euphoria, sedation, gastrointestinal motility, and respiratory depression. The most potent opioids have a particularly high affinity for μ-opioid receptors (19, 20). For example, affinity constants, *K*_*i*_, are 0.05 for carfentanil, 0.37 for hydromorphone, and 1.35 for fentanyl (the lower the value of *K*_*i*_, the higher the affinity for the receptor). In contrast, low affinity is observed for oxycodone (25.9) and methadone (3.4) (21). Additionally pure fentanyl is 224 times more potent than morphine and ~30–50 times more potent than heroin, and carfentanil is 10,000 times more potent than morphine (Figure 2) (3, 22). Potency refers to the concentration, or dose of a drug required to produce a defined effect [usually the concentration of the drug that produces 50% of the maximal possible effect (EC_50_)]. This is frequently confused with efficacy, which is the maximum effect attainable from the drug, i.e., once this magnitude of effect is reached, increasing the dose will not increase the magnitude of the effect (23).

Nitazenes (benzimidazole-opioids) are a class of synthetic opioids with non-fentanyl–based structural templates. Some that have been identified to date include etonitazene, isotonitazene, flunitazene, metonitazine, protonitazene, and 5-aminoisotonitazene (24, 25). These agents were initially developed for pain relief but have never been approved for use in humans. In general, nitazenes are about 10 to 40 times stronger than fentanyl (24, 26). The number of nitazene-related deaths has increased steadily over the past few years and they are considered an emerging threat. They are often not included in standard toxicology panels, so their prevalence is not well understood (27). Since 2019 there have been ~2,400 reports of illicit nitazenes to the US National Forensic Laboratory Information System (28).

Fentanyl is approved by the US Food and Drug Administration (FDA) and commonly used to treat pain in patients with cancer and also for other non-cancer–related clinical scenarios such as anesthesia and perioperative analgesia. The majority of OIRD deaths associated with fentanyl in the US are attributed to illicit manufacturing of fentanyl rather than prescription drug misuse. Compared to heroin, fentanyl is significantly cheaper and easier to synthesize and its higher potency makes it easy to hide and transport, reducing the risks to those involved in the illegal sale and distribution of drugs (29). Wholesale heroin costs in 2017 were ~$60,000 per kg, whereas fentanyl was much cheaper at ~$3,500 per kg (30). Fentanyl is frequently sold as counterfeit opioid pain pills made to look exactly like oxycodone, oxycodone/acetaminophen, or hydrocodone/acetaminophen tablets. A particular danger arises when fentanyl is added to cocaine or methamphetamine, as the risk of OIRD is very high, owing to the fact that these users are likely to be opioid-naive and therefore present a very high risk of overdose and death (29). Also, the end user of any drug not obtained from a pharmacy may encounter adulterated or counterfeit drugs where fentanyl is present without them knowing. In a study of a million urine specimens collected from patients during routine care, prevalence of non-prescribed fentanyl positivity among all cocaine-positive samples increased by 1,850% from 2013 to 2018 (from 0.9 to 17.6%) and by 798% among methamphetamine-positive samples (from 0.9 to 7.9%) (31). US Customs and Border Protection seized 14,700 tons of fentanyl illegally entering the country in 2022, an increase from 4,800 tons in 2020. In contrast, heroin seizures were 1,900 tons in 2022, a decrease from 5,800 tons in 2020 (32). From 2016 to 2020, the number of fentanyl drug trafficking offenses increased 1,946%, whereas the number of heroin and oxycodone offenders decreased by 33.2 and 47.1%, respectively. The only other trafficked drug that shows an increased number of offenders over this time period is methamphetamine (an increase of 13.9%) (33). The number of fentanyl drug-related offenses has continued to rise annually, with a 435% increase observed between 2018 and 2022 (34).

## Xylazine

Xylazine is a major adulterant of cocaine, heroin, and methamphetamine and frequently mixed with fentanyl (36, 37). It is available for veterinary use as an analgesic, sedative, and muscle relaxant. It is referred to as tranq, tranq-dope, sleep-cut, Philly dope, and zombie. Xylazine has the same target as the antihypertensive drug clonidine and the muscle relaxant tizanidine. It acts at the α-2-adrenergic receptor, causing CNS and respiratory depression, hypotension, hypothermia, and bradycardia similar to the effect of opioids. Users can develop dependence and withdrawal. When injected, severe, necrotic skin ulcerations may develop, sometimes in areas away from the injection site. These ulcerations are different from the infections often associated with drug injections (38). Its sedative effects allow those involved in the illegal sale and distribution of drugs to decrease the amount of opioid in the mixture and yet produce similar effects in the user, making the drug mixture more profitable for traffickers and dealers. However, as xylazine is not an opioid it is unaffected by naloxone, the main treatment for an opioid overdose (21). In addition, routine toxicology screens do not detect xylazine. This makes OIRDs due to combinations of opioids and xylazine particularly dangerous and life-threatening. Xylazine withdrawal symptoms cannot effectively be managed by methadone, buprenorphine, or naltrexone, the standard treatments used for OUD (36, 38). Because it is a veterinary drug, there is limited information about its pharmacokinetics in humans and how to manage overdose cases. In 2023, the Director of the Office of National Drug Policy Control officially designated fentanyl adulterated or associated with xylazine as an emerging threat to the US (39). Some states are planning to or have listed xylazine as a controlled drug (e.g., Ohio, Pennsylvania, West Virginia, and Pennsylvania), although it is not currently a controlled substance under the Federal US Controlled Substances Act (40).

## Treatment of OIRD

The primary cause of death in OIRDs is cardiac arrest secondary to respiratory arrest and asphyxia due to the opioid binding to the opioid receptors in the CNS (43). Initial symptoms of OIRD range from lethargy, depressed consciousness, pupillary miosis, to generalized CNS depression, followed by more serious conditions such as unconsciousness, seizures, and respiratory distress (shallow breathing, hypopnea, or bradypnea) leading to hypoxia (19). When hypoxia occurs, rapid treatment is required; brain damage may occur within 3–6 min of oxygen deprivation, followed by bradycardia and other cardiac dysrhythmias, cardiac arrest, and death shortly after. Therefore, first responders or caregivers have a very narrow treatment window following the onset of hypoxia during which to prevent an OIRD death (44, 45). The symptoms of opioid toxicity only occur when the product enters the bloodstream via ingestion, injection, or inhalation and cannot be experienced by touching the product.

### Naloxone

Treatment of OIRDs involves reversal of respiratory depression through administration of naloxone coupled with airway management and continuous assessment of oxygenation and ventilation (46, 47). Naloxone is an antagonist that competes for μ-, κ,- and δ-opioid receptor sites (48), thereby reversing the effects of opioids including respiratory depression, sedation, and hypotension (19). To successfully treat an OIRD, naloxone has to reach the opioid receptors as soon as possible and in sufficient concentration to displace the opioid from >50% of the receptors (21).

Current US-approved naloxone formulations include naloxone nasal sprays, prefilled naloxone injection devices for intramuscular (IM) or subcutaneous use, and generic naloxone for intravenous (IV), IM, or subcutaneous delivery (Table 1). Naloxone nasal spray (Narcan) is a prefilled nasal spray unit designed to deliver either 2 or 4 mg (44.2% bioavailable) of naloxone hydrochloride into the nasal cavity of a patient with a suspected opioid overdose (14). A prefilled nasal spray unit with a higher concentration of naloxone is also available (Kloxxado), designed to deliver 8 mg of naloxone (41.6%/47.6% bioavailable) (15). In addition to these, there are now generic naloxone nasal sprays (4 mg naloxone) available. The US FDA approved 4-mg intranasal (IN) naloxone for over-the-counter, non-prescription use in 2023 as a response to the need to reduce opioid-related deaths (49). A 3-mg IN dose was also recently approved for non-prescription use (50). Naloxone is also available as an IM injection (Zimhi) using a custom-designed injection device, similar to epinephrine injectors (EpiPen) for severe allergic reactions. An earlier IM autoinjector (Evzio, dispensing a 2-mg dose) was available but has been withdrawn for commercial reasons (51). Zimhi is a prefilled syringe containing 5 mg/0.5 mL naloxone for IM or SC administration. The syringe is housed within an opaque plastic case that incorporates a controllable plunger, flanges to support the fingers, a window for observing naloxone solution, a removable needle cover, and an extendable needle guard. It is designed for injection into the anterolateral aspect of the thigh, through clothing if necessary (16).

**Table 1:** Pharmacokinetic parameters of intranasal vs. intramuscular formulations of emergency use naloxone.

	**Intranasal (Narcan)** **(****14****)**		**Intranasal (Kloxxado)** **(****15****)**		**Intramuscular (Zimhi) (16)**
	**4 mg^a^**	**8 mg^b^**	**8 mg**		**5 mg/0.5 mL**
*N*	29	29	24	23	14
*C*_max_, ng/mL	4.83 (43)	9.70 (36)	12.3 (55.4)	12.8 (37.0)	17.2 (44)
*t*_max_, h	0.50 (0.17–1.0)	0.33 (0.17–1.00)	0.25 (0.10–1.0)	0.25 (0.10–1.0)	0.25 (0.17–0.52)
AUC_0 − inf_, ng·h/mL	7.95 (37)	15.5 (23)	16.7 (31.9)^c^	19.0 (32.7)^d^	26.6 (21.2)
*t*_1/2_, h	2.08 (30)	2.10 (32)	2.69 (69.9)	1.76 (39.7)^d^	1.5 (15.2)

^a^1 spray in 1 nostril.

^b^2 sprays, 1 in each nostril.

^c^n = 15.

^d^n = 19.

AUC_0 − inf_, area under the plasma concentration-time curve from time 0 extrapolated to infinity; C_max_, maximum plasma concentration; t_1/2_, apparent terminal elimination half-life; t_max_, time to maximum concentration. Mean (%CV) for all parameters except t_max_, which is median (range).

Naloxone is used intravenously in the emergency department for the treatment of OIRDs where it is dose titrated to reverse an overdose while minimizing acute withdrawal symptoms. However, as the opioid crisis has evolved, treatment has transitioned primarily from emergency department to first responders (emergency medical services, police, friends, and family). First responders were unwilling or unable to use IV administration as it is not practical given the equipment, supplies, and training required, and is more subject to human error, so nasal sprays expressly designed for rapid and easy administration have become the most common way of treating an overdose outside of the emergency department (52). These are fixed-dose devices and cannot be titrated.

**Figure 3:**
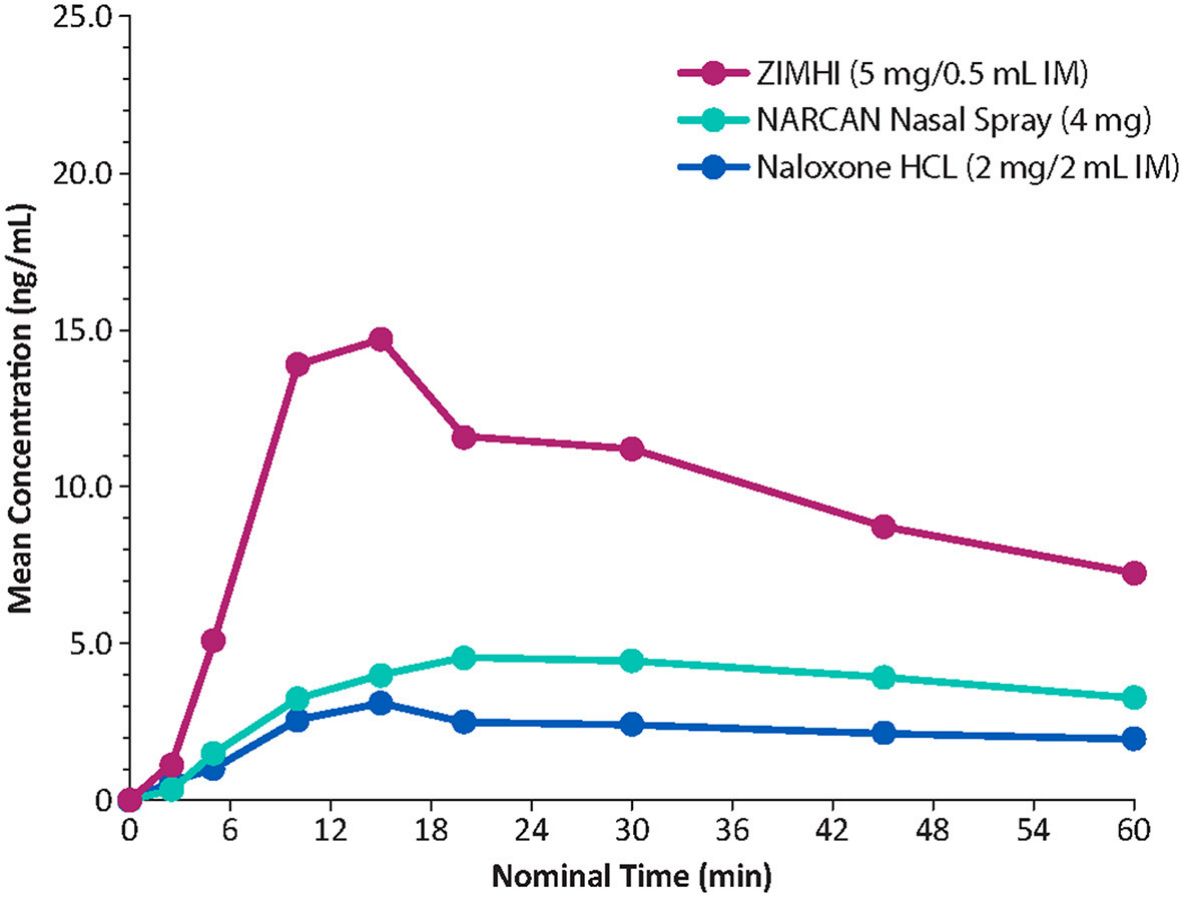
Comparison of mean plasma concentrations of naloxone over time in healthy adults. IM, intramuscular. Redrawn from Moss RB et al. (35) ^©^2019 Ronald B. Moss, an open-access article distributed under the Creative Commons Attribution License (CC BY).

Onset of action of naloxone is generally quickest (within 2 min) with IV administration, but IV dosing requires administration by medical personnel (e.g., emergency medical technicians/paramedics/nurses). In contrast, IM or subcutaneous administration can be performed easily and rapidly by family members, caregivers, or minimally trained first responders (e.g., police officers/firefighters) with expected onset of action between 2 and 5 min (19). IN administration has a slower onset of action than that observed with IV administration. In a comparative analysis of the pharmacokinetic profiles of Zimhi and the other currently available emergency naloxone formulations, Zimhi had a higher maximum observed plasma concentration (*C*_max_) and a greater area under the plasma concentration-time curve (AUC) than both the 4-mg IN Narcan and 2-mg/2-mL IM (generic naloxone) doses (Figure 3) (35).

A single 5-mg/0.5-mL Zimhi injection has greater bioavailability than 2 doses (8 mg total) of Narcan (Table 1) (35, 53). Zimhi administration therefore results in more rapid and higher systemic levels of naloxone than lower-dose IM/subcutaneous and IN formulations. Zimhi is not the only high-dose naloxone available for rapid administration when treating an overdose outside of the emergency room. Kloxxado nasal spray provides an 8-mg dose of naloxone (15). The IN 8-mg dose achieves more than twice the *C*_max_ of a 4-mg dose of Narcan in a median of half the time (15 vs. 30 min).

**Figure 4:**
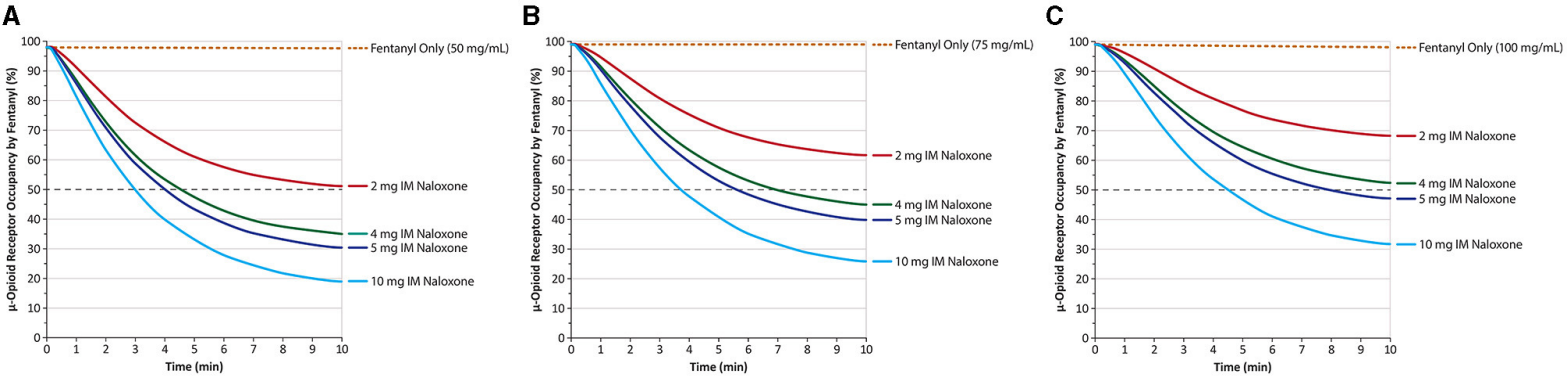
Predicted μ-opioid receptor occupancy by fentanyl after peak fentanyl plasma concentrations of 50 ng/mL **(A)** 75 ng/mL **(B)** and 100 ng/mL **(C)**. IM, intramuscular. Naloxone was given at time 0, and receptor occupancy was simulated based on known pharmacokinetic parameters of IM naloxone. Dashed gray line marks 50% receptor occupancy. Redrawn from Moss et al. (41) ^©^2022, Ronald B. Moss, This is an open access article distributed under the Creative Commons Attribution License (CC BY).

The implications of these differences for treating OIRDs have been investigated in preclinical studies and systems pharmacology modeling of the relationship between plasma naloxone levels and μ-opioid receptor occupancy. One study in rhesus monkeys found a direct correlation between the plasma levels achieved with higher doses of IM naloxone and the degree of μ-opioid receptor blockade observed in the basal ganglia and thalamus with an 0.06-mg/kg naloxone IM dose producing a mean 56% occupancy by naloxone of the μ-opioid receptors in the basal ganglia and 47% in the thalamus (54). Doses of 0.14 and 0.28 mg/kg increased the percentage of occupied receptors to 74 and 75% in the basal ganglia and 65 and 74% in the thalamus, respectively. A reduction to ≤ 50% μ-opioid receptor occupancy by fentanyl is associated with clinical reversal of opiate toxicity (55, 56). Higher doses of naloxone might be expected to reverse an overdose more effectively (57). To test this in a human clinical study is not possible due to the ethical issue of inducing an OIRD in participants, but pharmacokinetic modeling of naloxone-fentanyl competition at the μ-opioid receptor indicates that higher doses of naloxone are associated with more rapid decreases in μ-opioid receptor occupancy by fentanyl. At a mid-range fentanyl exposure of 50 ng/mL—with a predicted μ-opioid receptor occupancy of 97%—a 2-mg IM dose of naloxone is predicted to reduce μ-opioid receptor occupancy to 50% in 13.54 vs. 4.54 min with a 4-mg dose, 4 min with a 5-mg dose, and 3.04 min with a 10-mg dose (Figure 4A) (41).

Although a similar dose response is predicted at a fentanyl exposure level of 75 ng/mL (Figure 4B), the 2-mg IM naloxone dose does not reduce μ-opioid receptor occupancy by fentanyl to 50% within 10 min, whereas doses of 4, 5, and 10 mg reduced μ-opioid receptor occupancy to < 50% in 7.06, 5.68, and 3.76 min, respectively. At an even higher fentanyl exposure level of 100 ng/mL (Figure 4C), neither 2- nor 4-mg IM doses reduce μ-opioid receptor occupancy by 50% within 10 min, whereas 5 and 10 mg reduced them to < 50% in 8.02 and 4.54 min, respectively (41).

Because an overdose has to be treated as soon as possible in order to avoid death or CNS damage, it is vital that sufficient naloxone reaches the opioid receptors as soon as possible and in sufficient concentration to displace the opioid from >50% of the receptors in order to reverse the overdose (21).

These simulations, as well as feedback from first responders that multiple doses of traditional naloxone products are increasingly being required to treat OIRDs, suggest emergency naloxone [2 mg IM, 4 mg IN, 8 mg IN (equivalent to 4 mg IM)] may need to be administered in multiple doses for rapid reversal of fentanyl overdose at exposure levels capable of causing respiratory depression and death (41, 58–61). Thus, administration of higher doses (e.g., 5 and 10 mg IM) of naloxone has the potential to produce greater and more rapid antagonism of opioids at the μ-opioid receptor level and a swifter reversal of opioid toxicity (46, 61). It is important to note that other, external issues such as the time between naloxone doses, the drug combination used by the patient, and the utility of rescue or bag breathing/CPR are all very significant factors in reversing an OIRD.

Nitazenes are synthetic opioids so naloxone should be able to reverse an overdose. However, their high potency means that more and higher doses may be needed in a similar way as in the treatment of fentanyl OIRDs (24, 27, 28). In a cohort study of adults admitted to emergency departments with a presumed OIRD it was found that those patients (*n* = 9) who tested positive for brorphine [a potent, non-fentanyl–derived synthetic opioid (62)] or various nitazenes required in-hospital doses of naloxone with a cumulative dose of 4.4 mg compared to those in the fentanyl-only group (*n* = 11) with a cumulative dose of 6.41 mg. However, the non-fentanyl group was administered a statistically significantly higher number of in-hospital naloxone boluses compared with the fentanyl group, which suggests that brorphine and nitazenes may be more potent than fentanyl (63). As with synthetic opioids in general, better evidence is needed about the optimal naloxone doses, the delay between doses, and how many doses are ideally needed for treating nitazene overdoses (24, 27, 28).

The α_2_-adrenergic receptor agonist xylazine produces similar pharmacologic effects to opioids and may act in synergy with them (37). Because it is not active at opioid receptors, naloxone is thought to have no effect on xylazine poisoning (36, 64). In a patient experiencing a xylazine-adulterated OIRD, however, naloxone will still reverse the depression due to the opioid but will leave the depressive effect of the xylazine unaffected. For these patients, hospitalization is particularly vital as reversing xylazine intoxication will require various treatments and supportive care such as administration of IV fluids, intubation, and potentially cardiac catheterization (36). As it is impractical for the lay population and many first responders to know if a patient used a product containing xylazine, all patients with OIRDs should be taken to the hospital for evaluation and appropriate follow-up care.

### Nalmefene

Although this paper concentrates on naloxone, nalmefene, a μ-opioid receptor antagonist, should also be mentioned as a new treatment for OIRDs. It was approved by the FDA in 2023 as an IN spray (Opvee, Indivior Inc.) and used in the same way as naloxone nasal spray (65). Each dose contains 2.7 mg of nalmefene. Further nalmefene doses can be given every 2–5 min. Nalmefene was previously approved in 1995 as an injectable but was withdrawn for business reasons in 2008. Nalmefene has a longer half-life than naloxone (11.4 vs. ~2 h for IN naloxone), which may aid efficacy (66, 67). A disadvantage is that the longer half-life may result in longer periods of withdrawal symptoms (67).

## Concerns over high-dose naloxone

The approval and availability of high-dose formulations of naloxone for emergency treatment of opioid OIRDs are a welcome addition to a first-responder's toolkit; however, some concerns have been voiced about the necessity for their use. The pushback is largely encountered in 2 general areas. First, that there is no need for them, suggesting that data concerning the number of naloxone doses administered when treating OIRDs are lacking and have too many confounding factors (concomitant sedating drugs and adulterants) to indicate that standard dose naloxone is not sufficient to reverse the overdose (68). Second, that adverse effects associated with the use of high-dose naloxone formulations may precipitate “too severe” of a withdrawal, which may act as a deterrent to use by patients and caregivers (68, 69). Currently the authors have not found there to be evidence that indicates, on face value, that higher naloxone doses directly result in prolonged withdrawal. Patient perceptions, as assessed in a survey of 1,152 patients entering treatment for OUD, showed that 48.4% of respondents had no preference, 35.9% preferred a higher-dose naloxone formulation, and 10.9% preferred a standard dose if experiencing an OIRD. Similar preferences were found in community responders and in people administering naloxone to others. Respondents who had stated they preferred the high-dose formulations, however, had greater odds of having been exposed or suspected of having been exposed to fentanyl, consistent with greater knowledge of fentanyl's risks (70). In a 2022 study it was shown that 4-mg IN naloxone spray was thought to be not enough by members of the public that had, in the recent past, administered naloxone. The majority (87%) felt more confident in the efficacy of the 8-mg IN spray and 76% preferred to carry that over the 4-mg dose. The authors suggest this may be because of the participants' belief in the presence of fentanyl and other synthetic opioids in their community, that the 8-mg spray may work faster, and that they would need fewer sprays to reverse an OIRD (71).

### Precipitation of severe withdrawal

Adverse effects after the use of naloxone to reverse an OIRD in someone who is physically dependent are given in Table 2. There is a risk of vomiting and aspiration that is rarely fatal (72). In general, although these symptoms are undoubtedly unpleasant for the patient, these overdose effects are rarely life-threatening (59, 73). In fact, they should be considered of lesser importance than treating the OIRD, of which the primary symptoms are respiratory depression, unconsciousness, bradycardia, and hypothermia that are frequently fatal and are reversible with naloxone administration (59, 74). Reversing respiratory depression and restoring breathing is paramount. There is currently no objective withdrawal scale that could assess the degree of withdrawal correlated with naloxone dose nor how it might correlate with the opioid dose. There has also been concern that the severity of the precipitated withdrawal may correlate with the likelihood of the patient returning to substance use (68, 69). However, there is no published evidence to support this.

**Table 2:** Acute withdrawal adverse events associated with naloxone use treatment of opioid use disorder (14, 42).

• Body aches • Fever (pyrexia), chills • Sweating (hyperhidrosis) • Runny nose • Sneezing • Piloerection • Yawning • Weakness (asthenia) • Shivering or tremor • Nervousness, restlessness, or irritability • Diarrhea, nausea, vomiting • Abdominal cramps • Increased blood pressure, tachycardia • Aggressive behavior

### Pulmonary edema

Naloxone-induced non-cardiogenic pulmonary edema is a rare event that can occur following naloxone administration for reversing an opioid overdose (75–78). A proposed mechanism is that naloxone, by producing a rapid increase in catecholamines, produces an adrenergic crisis (79, 80), causing shifts in blood volume from the systemic bed to the pulmonary bed, thereby increasing permeability and inducing pulmonary edema (81). There is concern that higher doses of naloxone as provided by Kloxxado or Zimhi or by multiple Narcan doses may produce a higher risk of edema (75, 82). A 2020 study of 1,831 patients treated with naloxone for opioid overdose showed that pulmonary edema occurred in 1.1% and that those given out-of-hospital doses >4.4 mg were more likely to have a pulmonary complication (42 vs. 26% absolute risk) (83). In a recent retrospective study of patients (*n* = 639) treated for opioid overdose by emergency managements services or in the emergency department, it was found that 13 (2.0%) were diagnosed with pulmonary complications and that there was no significant correlation by naloxone dose (≤ 2 mg, >2 to ≤ 4 mg, and >4 mg) or route of administration, and the administration of high-dose naloxone was not associated with longer hospital stays (82). The causality of pulmonary complications and its relationship to overdose drug, dose, and naloxone dose needs to be examined further.

### Need for higher naloxone doses

The higher potency of fentanyl and fentanyl analogs such as carfentanil, compared to that of heroin, often requires multiple doses of naloxone, which suggest there is a need for higher-dose naloxone (84–86). The label for each of the emergency naloxone treatments gives 2–3 min as the time to wait between doses, and that further additional doses should be given until emergency medical assistance arrives (14–16). A patient may revive only to fall back into respiratory depression as the half-life of naloxone is short (~1.5–2.5 h) and naloxone levels may fall below levels at which the μ-opioid receptors are sufficiently blocked. This may lead to rebinding of opioid still present in the patient, requiring another naloxone administration. In general, the quality of available naloxone dosing data used to reverse an overdose in the population is often unsatisfactory as the number of doses are often not reported, or the total dose is recorded with no account of whether this represents the total given as prescribed 2–3 min apart or whether it reflects more than 1 initial dose.

In addition to these concerns, the presence of a higher-dose formulation may suggest to patients that they are the better, or the best, option, which may lead to reduction in use of lower-dose formulations. This might deter people from using other options, even if these are the only ones available. At this point in the opioid overdose epidemic, all naloxone should be considered good naloxone. While providing higher-dose naloxone may produce the best outcomes, communities should not let perfect be the enemy of good. Any naloxone product is better than none and the need for greater access to naloxone is paramount.

### Other issues

A common issue with injectable naloxone is the needle. This is frequently cited as a major concern for both first responders (particularly police) and caregivers. It stands to reason that people with substance use disorder who used a syringe to take opioids are unlikely to be concerned about an injection device. Many people when they hear that Zimhi is an injection may assume, incorrectly, that it is a syringe with a vial that requires liquid to be drawn up and a standard injection procedure to be followed. This gives rise to worries about administering the wrong amount or making an error because of being in the high stress situation of an OIRD. In reality, Zimhi is very easy to use, as demonstrated in usability studies (87). There is a needle shield that prevents injury after use, which is important as the potential for an accidental needle stick is often cited as an issue with syringe and needle products. Education should convert users to overcome this worry. A demonstration of how to use it, and giving people the chance to handle the Zimhi device should allay fears. Like Kloxxado 8-mg nasal spray, Zimhi is ready to use, effective, and is the most concentrated and has the highest dose available on the market in a single-use device. Both are as easy to use and carry as the more commonly available 2- or 4-mg Narcan sprays.

## Real-world issues faced when responding to OIRDs

Despite the widespread publicity of the opioid/fentanyl epidemic, many people are unaware of naloxone and how to use it. Education is important not only in informing people how to treat emergency OIRDs but also how to obtain naloxone. Families of people who use drugs may not want to go to the pharmacy to obtain naloxone because of the stigma associated with OUD or worry that the health insurance company may find out, with negative effects on their coverage or their employment. In many cases, naloxone is administered by families, bystanders, or fellow users and first responders are not called. Police, in particular, are often not welcome because of the potential criminality of drug offenses (88), despite “Good Samaritan laws” in 47 states that provide legal immunity for friends, family, and other bystanders for someone who needs medical aid for an overdose (89). Education is required to make sure that a 911 call is made before or immediately after administering naloxone because, although the patient may have been revived, they may overdose again later because of the short half-life of naloxone and the long-lasting potency of fentanyl and its analogs (46). There is also the potential need for acute supportive as well as ongoing care. As many patients may suffer from a polysubstance use disorder, the need for care in a hospital setting for drugs other than opioids (e.g., xylazine and methamphetamine) will be necessary.

The CDC has guidelines specifying that patients who are taking opioids and who have overdose risk factors should be offered a naloxone prescription or a co-prescription and should be instructed on how to use it in order to reduce OIRD deaths (90). Overdose risk factors include taking high doses of opioids (≥50 morphine mg equivalents per day), being prescribed benzodiazepines in addition to opioids, receiving treatment for OUD (i.e., taking methadone or buprenorphine), having medical conditions that may increase the risk of overdose such as chronic obstructive pulmonary disease or obstructive sleep apnea, having excessive alcohol use or other substance use disorder, and being aged 65 years or older. Currently, there are 100 prescriptions for high-dose opioids for every 1.5 naloxone prescriptions (90).

Although naloxone accessibility has dramatically risen in the past 20 years, its distribution from primary access points (such as in community pharmacies) remains low (91). It is important to note that naloxone not only serves as a life-saving medication, but may act as the mechanism to bridge a patient into treatment. While no set number of OIRDs is required to achieve a given patient's journey to recovery, providing a patient with the opportunity to use evidence-based, medication-assisted treatment for OUD (MOUD) such as buprenorphine or methadone may be an overlooked yet critical benefit of naloxone use. Unfortunately, many who suffer from substance use disorder do not feel they need help, or do not consider themselves at risk (92). Furthermore, many factors previously discussed (e.g., stigma and cost) compound this issue. It is essential that part of any naloxone rescue includes a warm handoff to a treatment provider in the patient's local community. With the recent elimination of the barriers on buprenorphine prescribing, it is possible that a greater number of prescribers, including pharmacists in select jurisdictions, may be able to further assist patients in this regard (93). Further education and advocacy are required though on not just naloxone use, but MOUD, in order to provide greater acceptance by providers, policy makers, and the public as a safe and effective treatment option.

There is currently no national system providing data on the number of naloxone doses required to treat fentanyl-derivative OIRDs. The Overdose Detection Mapping Application Program (ODmap www.odmap.org) provides real-time data on OIRDs (94, 95). It is a data collection and agency administration interface only available to government (federal, state, local, or tribal) agencies serving the interests of public safety and public health. This is a useful tool for law enforcement and first responders. The data provide the location, whether the overdose was fatal or non-fatal, and number of doses required for each recorded overdose. Disadvantages are that it is not mandatory for all states to enter data, and the data entered are often not complete and, in some cases, overstretched EMS departments lack personnel to enter the data.

Naloxone can be obtained by prescription and is now available over the counter at pharmacies but also through charities, non-profits, harm reduction groups, and community programs. Individuals need to be educated on the importance of obtaining naloxone and where it can be procured. It is vital that all FDA-approved emergency use naloxone products in both IN and IM formulations and at all doses are made freely available going forward, with the goal of halting this horrific epidemic that has claimed so many lives.

## Author contributions

AD: Writing—original draft, Writing—review & editing. TF: Writing—original draft, Writing—review & editing. JC: Writing—original draft, Writing—review & editing. DC: Writing—original draft, Writing—review & editing. CC: Writing—original draft, Writing—review & editing. JLG: Writing—original draft, Writing—review & editing. JCG: Writing—original draft, Writing—review & editing. JP: Writing—original draft, Writing—review & editing. DR: Writing—original draft, Writing—review & editing.
